# CDC Clinical Guidelines on the Use of Doxycycline Postexposure Prophylaxis for Bacterial Sexually Transmitted Infection Prevention, United States, 2024

**DOI:** 10.15585/mmwr.rr7302a1

**Published:** 2024-06-06

**Authors:** Laura H. Bachmann, Lindley A. Barbee, Philip Chan, Hilary Reno, Kimberly A. Workowski, Karen Hoover, Jonathan Mermin, Leandro Mena

**Affiliations:** ^1^Division of STD Prevention, National Center for HIV, Viral Hepatitis, STD and TB Prevention, CDC; ^2^Department of Medicine, Brown University, Providence, Rhode Island; ^3^Department of Medicine, Washington University School of Medicine in St. Louis, St. Louis, Missouri; ^4^Department of Medicine, Emory University, Atlanta, Georgia; ^5^Division of HIV Prevention, National Center for HIV, Viral Hepatitis, STD and TB Prevention, CDC, Atlanta, Georgia; ^6^National Center for HIV, Viral Hepatitis, STD and TB Prevention, CDC, Atlanta, Georgia

## Abstract

No vaccines and few chemoprophylaxis options exist for the prevention of bacterial sexually transmitted infections (STIs) (specifically syphilis, chlamydia, and gonorrhea). These infections have increased in the United States and disproportionately affect gay, bisexual, and other men who have sex with men (MSM) and transgender women (TGW). In three large randomized controlled trials, 200 mg of doxycycline taken within 72 hours after sex has been shown to reduce syphilis and chlamydia infections by >70% and gonococcal infections by approximately 50%.

This report outlines CDC’s recommendation for the use of doxycycline postexposure prophylaxis (doxy PEP), a novel, ongoing, patient-managed biomedical STI prevention strategy for a selected population. CDC recommends that MSM and TGW who have had a bacterial STI (specifically syphilis, chlamydia, or gonorrhea) diagnosed in the past 12 months should receive counseling that doxy PEP can be used as postexposure prophylaxis to prevent these infections. Following shared decision-making with their provider, CDC recommends that providers offer persons in this group a prescription for doxy PEP to be self-administered within 72 hours after having oral, vaginal, or anal sex. The recommended dose of doxy PEP is 200 mg and should not exceed a maximum dose of 200 mg every 24 hours.

Doxy PEP, when offered, should be implemented in the context of a comprehensive sexual health approach, including risk reduction counseling, STI screening and treatment, recommended vaccination and linkage to HIV PrEP, HIV care, or other services as appropriate. Persons who are prescribed doxy PEP should undergo bacterial STI testing at anatomic sites of exposure at baseline and every 3–6 months thereafter. Ongoing need for doxy PEP should be assessed every 3–6 months as well. HIV screening should be performed for HIV-negative MSM and TGW according to current recommendations.

## Background

Incidence of sexually transmitted infections (STIs) caused by *Neisseria gonorrhoeae*, *Chlamydia trachomatis*, and *Treponema pallidum* continues to increase in the United States (*1*). Novel approaches are needed to address the STI epidemic, especially for populations disproportionately affected (*2*). Postexposure prophylaxis (PEP) involves taking a medication to prevent an infection after a possible exposure and is a common strategy for prevention of HIV and other infections. PEP is a form of chemoprophylaxis and is distinct from pre-exposure prophylaxis (PrEP), which involves taking a medication before exposure occurs. Doxycycline is used as PrEP or PEP to prevent infections such as malaria and Lyme disease (*3*) but, until recently, has not been used to prevent STIs. These CDC recommendations for an ongoing, patient-managed STI prevention strategy include a novel approach to PEP that provides the patient a prescription allowing them to have doxycycline for self-administration as soon as possible after sex to prevent syphilis, chlamydia, and gonorrhea.

Doxycycline, a broad-spectrum tetracycline antimicrobial, is well absorbed and tolerated, with a half-life of approximately 12 hours (*4*). Adverse effects most associated with doxycycline include photosensitivity and gastrointestinal symptoms including esophageal erosion and ulceration (*5*). Most adverse effects resolve with discontinuation of the medication. Doxycycline is the recommended treatment regimen for chlamydia and an alternative treatment for syphilis in nonpregnant patients with severe penicillin allergy or when penicillin is not available (*6*). Although currently not a recommended treatment for gonorrhea because of elevated antimicrobial resistance, doxycycline remains effective against many strains of *N. gonorrhoeae* in the United States (*7*).

In 2015, the treatment arm of a randomized clinical controlled trial studied the use of oral doxycycline hyclate 100 mg daily as STI PrEP among 30 men who have sex with men (MSM) with HIV receiving antiretrovirals (*8*). At 48 weeks, persons who were assigned daily STI PrEP had a 73% reduction in bacterial STI incidence compared with persons in the comparison arm demonstrated by decreases in chlamydia and syphilis but not gonorrhea. The 2021 CDC STI treatment guidelines included a systematic review of the available literature on STI PrEP and PEP and concluded that further studies were necessary to determine whether STI chemoprophylaxis would be an effective strategy for bacterial STI prevention (*6*). Since the study in 2015, there have been no new studies of STI PrEP, but three large randomized controlled trials among MSM and transgender women (TGW) studied STI PEP in the form of 200 mg of doxycycline taken after sex. These trials all demonstrated significant reductions in bacterial STI acquisition (syphilis, chlamydia, and gonorrhea). Between 2023 and 2024, certain countries have issued varying position statements on the practice of doxy PEP ranging from recommending against widespread use because of concerns about antimicrobial resistance (*9*) to conditional use for MSM and TGW at risk for syphilis (*10*) or on a case-by-case basis (*11*). This report presents CDC’s updated clinical guidelines for health care providers to inform MSM and TGW who have had a bacterial STI (specifically syphilis, chlamydia, and gonorrhea) diagnosed in the past 12 months about the use of doxy PEP for preventing these bacterial STI infections.

## Methods

The guidelines were developed by a multidisciplinary work group of CDC staff who are physicians with expertise in infectious diseases, STIs, HIV, and public health. A systematic literature review was conducted to inform the question: Does doxycycline taken after vaginal, anal, or oral sex decrease bacterial STIs (i.e., syphilis, chlamydia, and gonorrhea) compared with not taking doxycycline? Studies published through June 2023 using MEDLINE/PubMed and Embase were included. Studies that met inclusion criteria (i.e., randomized controlled trials, written in English, and evaluated doxy PEP as STI prophylaxis) were given a summary strength of evidence rating using the same approach as the U.S. Department of Health and Human Service’s Panel on Antiretroviral Guidelines for Adults and Adolescents (Table) (*12*). The Grading of Recommendations, Assessment, Development, and Evaluations evidence-to-decision framework was used to weigh the benefits and harms, values, acceptability, equity, and feasibility. Abstracts presented at major scientific meetings (i.e., Conference on Retroviruses and Opportunistic Infections and the STI and HIV World Congress) also were reviewed. Literature reviews also were conducted to address the question: Does long-term doxycycline use cause substantial harms such as the development of antimicrobial resistant pathogens, and dermatologic, gastrointestinal, neuropsychiatric, and metabolic side effects? Evidence was not graded. Further details regarding search strategies are available https://www.cdc.gov/std/treatment/guidelines-for-doxycycline.htm.

**TABLE:** Rating scheme for strength and quality of evidence supporting CDC recommendations

Strength of recommendations for the statement	Quality of evidence supporting a recommendation
A. Strong recommendation	I. One or more randomized trials with clinical outcomes and/or validated laboratory endpoints
B. Moderate recommendation	II. One or more well-designed, nonrandomized trials or observational cohort studies with long-term clinical outcomes
C. Weak recommendation	III. Expert opinion

In addition to the literature reviews, the National Association of County and City Health Officials held a virtual 2-day consultation December 5 and 6, 2022, during which multiple experts, stakeholders, and community members discussed doxy PEP, including benefits and potential harms of use. The meeting report (https://www.naccho.org/uploads/downloadable-resources/STI-Post-Exposure-Prophylaxis-with-Doxycycline-Report.pdf) was reviewed by the Doxy PEP Guidelines Development Workgroup, and findings from the consultation were considered during the development of these recommendations. A virtual bioethics consultation took place April 14 and May 1, 2023, and included bioethics experts, academic experts in infectious diseases and health equity, community advocates, and CDC staff. This consultation resulted in a publication (https://journals.lww.com/stdjournal/citation/9900/ethical_considerations_in_implementing_doxycycline.282.aspx), and opinions from individual experts were considered in the development of the guidelines and implementation plans.

Members of the work group provided names of potential reviewers, aiming for diversity of backgrounds and regions in the United States and expertise in infectious diseases, STI and HIV prevention, antimicrobial resistance, and therapeutics. Peer reviewers reviewed draft recommendations, responded to five specific questions, and provided additional comments. Reviewers disclosed any potential conflicts of interest and conflicts, if present, were resolved. The document was posted on October 2, 2023, for 45 days on the federal registry for public comment. Comments from peer reviewers and the public were addressed and the document revised as appropriate. Evidence and feedback were reviewed by the work group, and final recommendations were developed by CDC staff.

In this report, the terms MSM and TGW who have sex with men are used as they were defined by the studies conferring the evidence base for this guidance. However, the language to describe the populations of focus in this guidance conflates both gender identity, sexual orientation, and sexual behavior that might be separate and distinct for certain persons. In addition, the likelihood of infection with a bacterial STI is related to both behavior and the sexual networks within which the behavior occurs.

## Efficacy of Doxy PEP for Reducing Bacterial STIs

Four studies on the efficacy of doxy PEP for reducing bacterial STIs are summarized in this report. In the open-label extension phase of the French IPERGAY Study, doxycycline was evaluated as PEP among a single cohort of 232 HIV-negative MSM and TGW taking tenofovir disoproxil fumarate and emtricitabine as PrEP for HIV prevention. Participants were randomized to either take a single oral dose of 200 mg doxycycline, ideally within 24 hours and no later than 72 hours after having condomless anal or oral sex up to three times per week, versus no medication prophylaxis (*13*). The primary endpoint was occurrence of a first bacterial STI infection (syphilis, chlamydia, and gonorrhea) during a 10-month follow-up period. Persons who took doxy PEP had a reduced risk for acquiring chlamydia and syphilis of 70% (hazard ratio [HR]: 0.30; 95% CI = 0.13–0.70) and 73% (HR: 0.27; 95% CI = 0.07–0.98), respectively. No significant difference in gonorrhea infections was observed between the two groups (HR: 0.83; 95% CI = 0.47–1.47).

In 2022, a randomized open-label clinical trial (DoxyPEP) in San Francisco, California, and Seattle, Washington, evaluating 501 MSM and TGW with HIV infection (N = 174) or on HIV PrEP (N = 327) with a history of condomless sex with one or more male partner and one or more STIs during the previous 12 months found that oral doxycycline hyclate 200 mg self-administered ideally within 24 hours but no later than 72 hours after condomless anal, oral, or vaginal sex significantly reduced the incidence of chlamydia, gonorrhea, and syphilis (*14*). Reductions were noted in relative risk (RR) of gonorrhea among persons taking HIV PrEP (RR: 0.45; 95% CI = 0.34–0.65) and persons with HIV infection (RR: 0.43; 95% CI = 0.26–0.71); chlamydia (RR: 0.12; 95% CI = 0.05–0.25 and RR: 0.26; 95% CI = 0.12–0.57) and early syphilis (RR: 0.13; 95% CI = 0.03–0.59 and RR: 0.23; 95% CI = 0.04–1.29) also were reduced. The number needed to treat to prevent a quarterly visit with an incident STI was 4.7 in the PrEP cohort and 5.3 in persons living with HIV infection. Participants were advised to take no more than 200 mg of doxycycline every 24 hours. In the intervention arm, 86% reported taking doxycycline always or often and 71% reported never missing doxycycline within 72 hours of condomless sex.

Also in 2022, the French ANRS DOXYVAC study, which enrolled MSM on HIV PrEP for at least 6 months and who had at least one STI in the 12 months before enrollment, was stopped early because of intervention efficacy. This trial randomly assigned MSM to PEP with doxycycline monohydrate within 24–72 hours of sex (N = 332) or no doxy PEP (N = 170) and then to vaccination with 4CMenB (Bexsero) (N = 257) or no vaccine (N = 245). Participants were followed up to 96 weeks with the primary efficacy endpoint of impact of doxy PEP on time to first episode of syphilis or chlamydia. Initial results demonstrated that doxy PEP was associated with significant reductions in gonorrhea (adjusted hazard ratio [aHR]: 0.49; 95% CI = 0.32–0.76), chlamydia (aHR: 0.11; 95% CI = 0.04–0.30), and syphilis (aHR: 0.21; 95% CI = 0.09–0.47) (*15*).

The only trial conducted among cisgender women was an open-label 1:1 randomized trial of doxycycline 200 mg within 72 hours of sex versus standard of care conducted during 2020–2022 in 449 cisgender Kenyan women. It found no significant reduction in bacterial STIs (RR: 0.88; 95% CI = 0.60–1.29), chlamydia (RR: 0.73; 95% CI = 0.47–1.13), or gonorrhea (RR: 1.64; 95% CI = 0.78–3.47) (*16*). Syphilis efficacy could not be evaluated because there were only two early syphilis infections during the study. Although women assigned to doxy PEP reported event-driven dosing coverage in 78% of weekly surveys, hair studies found that doxycycline was detected in only 29% of participants in the doxycycline arm, suggesting that nonadherence might have been the reason for lack of efficacy; however, additional reasons for lack of efficacy, such as biologic differences, still need to be explored (*16*,*17*).

## Potential Harms from Doxycycline

### Clinical Adverse Events in Doxy PEP Trials

Three clinical trials of doxy PEP were found that reported on adverse events (*13*–*15*). In the IPERGAY study, gastrointestinal side effects were more commonly reported in the PEP group (53% versus 41%; p = 0.05) (*13*). In the DoxyPEP study, one grade 2 laboratory abnormality and five grade 3 adverse events occurred that were possibly or probably related to doxycycline. No serious adverse events were attributed to doxycycline. The observed difference in mean absolute annualized weight change adjusted for baseline weight was not statistically significant between the two groups. Eighteen participants discontinued the study early; five in the doxy PEP arm discontinued the medication because of intolerance or patient preference and 13 in the standard of care arm, including six who discontinued so that they could take doxy PEP outside of the study (*14*). In the DOXYVAC study, three persons (0.9%) discontinued doxy PEP because of gastrointestinal adverse events (n = 2) or fear of adverse events (n = 1) (*15*).

### Clinical Adverse Events from Other Uses of Doxycycline

To further examine data on daily longer-term (≥8 weeks) doxycycline use, a systematic literature review (https://www.cdc.gov/std/treatment/guidelines-for-doxycycline.htm) and meta-analysis were conducted (*18*). A total of 67 articles published during 1987–2022 were included in the review on clinical adverse events; half of the studies included doxycycline use for acne treatment (N = 13), malaria prophylaxis (N = 11), and rosacea treatment (N = 14). A meta-analysis conducted by CDC included 18 studies that enrolled generally healthy persons and that also had a placebo arm to evaluate any adverse events between participants in the doxycycline group versus the no doxycycline (placebo) group. The meta-analysis found an increased risk for persons on daily doxycycline experiencing gastrointestinal or dermatological adverse events compared with placebo. No significant differences were observed for any, severe, or neurologic adverse events. Participants were more likely to be unenrolled from clinical trials because of adverse events in the doxycycline group compared with the placebo group. Serious side effects were rare.

### Potential Resistance in Commensals and Co-Occurring Pathogens

Another potential concern about doxycycline PEP is facilitating antimicrobial resistance both in bacterial STIs and other common bacterial pathogens (e.g., *Staphylococcus aureus*). Data from the 12-month DoxyPEP follow-up period evaluating tetracycline resistance in *S. aureus* isolates found that those in the doxy PEP arm had a reduction of carriage of *S. aureus* overall of 14% (from 44% [187 of 428] to 31% [69 of 222]), but that those with *S. aureus* in their nares at 12-month follow-up had an increase in tetracycline-resistant *S. aureus* (from 5% [20 of 428] to 13% [28 of 222]). There was no change in carriage or resistance in the standard of care arm (carriage 48% [96 of 202] at baseline and 43% [28 of 65] at 12-month follow-up; resistance was 10% [21 of 202] at baseline and 5% [three of 65] at follow-up). Although limited by the low number of *N. gonorrhoeae* isolates with MIC data available (56 of 320), 24% (four of 17) of gonococcal isolates were tetracycline resistant at baseline compared with 11% (two of 19) of incident gonococcal isolates in the standard of care arm and 30% (six of 20) in the doxy PEP arm (*14*,*19*). Data from the ANRS DOXYVAC study found 100% of gonococcal isolates tested at baseline (N = seven) were tetracycline resistant (defined as MIC >0.5 mg/L or for high-level resistance, MIC>8 mg/L by Etest) (*20*,*21*). Among the gonococcal isolates recovered during follow-up in the doxy PEP arm, 67% (14 of 21) and 33% (seven of 21) demonstrated resistance and high-level resistance, respectively, versus 81% (30 of 37) and 19% (seven of 37), respectively, in the no-PEP arm (*15*). Limited phenotypic and genotypic testing of *C. trachomatis* strains failed to reveal significant trends in the development of resistance. Trends in methicillin-resistant *S. aureus* and extended-spectrum beta-lactamase–producing *Escherichia coli* colonization rates did not differ between study arms.

Other studies evaluating the association of doxycycline on antimicrobial resistance of non-STI pathogens are limited, but data have been reported for acne treatment and malaria prophylaxis. Studies have largely found no relation between daily doxycycline and doxycycline resistance in *Cutibacterium acnes*, *Staphylococcus epidermis*, or gastrointestinal pathogens causing diarrhea; however, lower doses of doxycycline were used in these studies (*22*,*23*). Few studies have examined the characteristics of *S. aureus* in persons taking daily doxycycline, but one study found that Panton-Valentine leukocidin-positive methicillin-sensitive *S. aureus* was more common in persons on daily doxycycline than persons taking other malarial prophylaxis, and all Panton-Valentine leukocidin-positive doxycycline-resistant methicillin-sensitive *S. aureus* isolates were found in persons taking doxycycline (*24*). The ability to draw conclusions from these studies is limited by the heterogeneity of studies and dosage and treatment duration compared with doxy PEP, and consideration should be given to monitoring other pathogens such as those that cause community-acquired pneumonia. There are no studies to date on long-term, intermittent use of doxycycline and the microbiome. Current data suggest overall benefit of the use of doxy PEP, but potential risks related to the development of resistance and changes in the microbiome will need to be monitored as these guidelines are implemented.

## Identifying Populations that Would Benefit Most from Doxy PEP

The goal of doxy PEP is for persons who would benefit most to have access to the intervention while minimizing antimicrobial use. Data from the DoxyPEP study indicate that persons in the study took, on average, an additional 43 doses of doxycycline to prevent an average of 1.3 bacterial STIs annually. Other studies have found that among MSM taking HIV PrEP, a subset of persons account for the majority of bacterial STIs. For example, in one study of 2,981 MSM, 25% of persons accounted for 76% of bacterial STIs (*25*). Although models indicate that a substantial number of MSM would need to use STI PEP or PrEP for population-level effectiveness, this effect would be most pronounced if focused on subpopulations with higher likelihood of STIs (*26*) compared with the broader MSM and TGW population (*27*,*28*). In a cohort of MSM and TGW and nonbinary persons assigned male sex at birth, prescribing doxy PEP for 12 months after an STI diagnosis was the most efficient strategy, averting 42% of STIs with the number needed to treat for 1 year to avert any STI of 2.2 (*29*).

## Recommendations

### Rationale

Because of increasing rates of bacterial STIs and the reported high efficacy for the reduction of STIs in the reviewed clinical trials, the potential benefits of doxy PEP are notable. Systematic reviews of potential harms appear low in the short-term and unknown but potentially concerning in the long-term. Overall, the intervention appears feasible and acceptable and will require a focused effort for equitable implementation. In considering all of these issues, and in review of studies of populations who would likely benefit most from an intervention to reduce bacterial STIs balancing efficacy and the risk for antimicrobial resistance, the following considerations are recommended.

### Populations

CDC recommends that providers should counsel gay, bisexual, and other MSM and TGW with a history of at least one bacterial STI (specifically syphilis, chlamydia, or gonorrhea) during the past 12 months about the benefits and harms of using doxy PEP (Box 1). Although not directly assessed in the trials included in these guidelines, doxy PEP could be discussed, using a shared decision-making approach, with MSM and TGW who have not had a bacterial STI diagnosed during the previous year but will be participating in sexual activities that are known to increase the likelihood of exposure to STIs. Although the pharmacokinetics of doxycycline and experience in treating bacterial STIs suggest that doxy PEP should be effective in other populations, clinical data to support doxy PEP in other populations (i.e., cisgender women, cisgender heterosexual men, transgender men, and other queer and nonbinary persons assigned female at birth) are limited. As a result, providers should use their clinical judgement and shared decision-making to inform use of doxy PEP with populations that are not part of CDC recommendations.

BOX 1 **CDC recommendations for use of doxycycline as postexposure prophylaxis for bacterial sexually transmitted infections prevention**

**Table:** 

Recommendation*	Strength of recommendation and quality of evidence^†^
• Providers should counsel all gay, bisexual, and other men who have sex with men (MSM) and transgender women (TGW) with a history of at least one bacterial sexually transmitted infection (STI) (specifically, syphilis, chlamydia or gonorrhea) during the past 12 months about the benefits and harms of using doxycycline (any formulation) 200 mg once within 72 hours (not to exceed 200 mg per 24 hours) of oral, vaginal, or anal sex and should offer doxycycline postexposure prophylaxis (doxy PEP) through shared decision-making. Ongoing need for doxy PEP should be assessed every 3–6 months.	**AI** **High-quality evidence supports this strong recommendation to counsel MSM and TGW and offer doxy PEP.**
No recommendation can be given at this time on the use of doxy PEP for cisgender women, cisgender heterosexual men, transgender men, and other queer and nonbinary persons.	**Evidence is insufficient to assess the balance of benefits and harms of the use of doxy PEP**

*Although not directly assessed in the trials included in these guidelines, doxy PEP could be discussed with MSM and TGW who have not had a bacterial STI diagnosed during the previous year but will be participating in sexual activities that are known to increase likelihood of exposure to STIs.

^†^ See Table.

### Administration and Dosage

If doxy PEP is prescribed, the provider should write the prescription for self-administration of the recommended dose of 200 mg of doxycycline (any formulation) to be taken as soon as possible within 72 hours after having oral, vaginal, or anal sex with a maximum dose of 200 mg every 24 hours. The prescription should account for enough doses on the basis of the person’s anticipated sexual activity until their next visit. Ongoing need for doxy PEP should be assessed every 3–6 months.

### Ancillary Clinical Services with Doxy PEP

Doxy PEP, when offered, should be implemented in the context of a comprehensive sexual health approach (Box 2), including risk reduction counseling, STI screening and treatment, recommended vaccination and linkage to HIV PrEP, HIV care, or other services as appropriate (*6*). Persons who are prescribed doxy PEP should undergo bacterial STI testing at anatomic sites of exposure at baseline and every 3–6 months thereafter. HIV screening should be performed for HIV-negative MSM and TGW according to current recommendations (*6*).

BOX 2 **Considerations for ancillary clinical services to provide to persons receiving doxycycline postexposure prophylaxis for the prevention of syphilis, chlamydia, and gonorrhea**

**Table:** 

At initial postexposure prophylaxis (PEP) visit
Screen and treat as indicated for sexually transmitted infections (STIs) (obtain nucleic acid amplification test for gonorrhea and chlamydia at anatomic sites of exposure and serologic testing for syphilis). For persons without HIV infection receiving HIV pre-exposure prophylaxis (PrEP), screen per CDC HIV PrEP guidelines (https://www.cdc.gov/hiv/pdf/risk/prep/cdc-hiv-prep-guidelines-2021.pdf). For persons without HIV infection not receiving HIV PrEP, consider screening for HIV infection every 3–6 months. Counsel on use of prevention strategies including condom use, consideration of reducing the number of partners, and accessing HIV PEP, PrEP or HIV treatment as indicated. Counseling should include: A discussion of the benefits and potential harms of doxycycline PEP including known side effects such as photosensitivity, esophagitis and esophageal discomfort, gastrointestinal intolerance (nausea, vomiting, and diarrhea), and the potential for the development of antimicrobial resistance in other pathogens and commensal organisms and changes in the microbiome and the unknown long-term effects that might cause. Guidance on actions to take to mitigate potential side effects including taking doxycycline on a full stomach with a full glass of liquid and avoiding lying down for 1 hour after taking doxycycline to prevent esophagitis. The need to take doxycycline exactly as individually prescribed and only for its intended purpose. Patients should not take more than 200 mg of doxycycline per 24 hours; doses should be taken as soon after sex as possible, but no later than 72 hours. Counsel on potential drug interactions including the importance of separating the doxycycline dose by at least 2 hours from dairy products, antacids, and supplements that contain calcium, iron, magnesium, or sodium bicarbonate. No clinically relevant interactions between doxycycline and gender-affirming hormonal therapy are likely. Because doxycycline interacts with other drugs, providers should review patient’s medication list, including over the counter medications, to assess for possible drug interactions. Provide enough doses of doxycycline to last until the next follow-up visit, based on individual behavioral assessment through shared-decision making.
At follow-up visits
Screen for gonorrhea and chlamydia at anatomic sites of exposure and syphilis every 3–6 months per CDC STI treatment guidelines recommendations for screening men who have sex with men and transgender women. For persons without HIV receiving HIV PrEP, screen per CDC HIV PrEP guidelines (https://www.cdc.gov/hiv/pdf/risk/prep/cdc-hiv-prep-guidelines-2021.pdf). For persons without HIV infection not receiving HIV PrEP, consider screening for STIs and HIV infection every 3–6 months. Assess for the need for HIV PEP and encourage the use of HIV PrEP. Confirm or encourage linkage to HIV care for persons living with HIV infection. Assess for side effects from doxycycline. Provide risk reduction counseling and condoms. Re-assess continued need for doxy PEP. Provide enough doses of doxycycline until next follow-up visit, based on individual behavioral assessment through shared-decision making.
Additional services to consider
Screen for hepatitis B and C infection; vaccinate against hepatitis B if susceptible. Administer other vaccines as indicated (mpox, hepatitis A, and human papillomavirus). Refer for comprehensive primary care, mental health services, substance use treatment, and other services as appropriate.

## Conclusion

Doxy PEP has demonstrated benefit in reducing incident syphilis, chlamydia, and gonorrhea in certain populations and represents a new approach to addressing STI prevention in MSM and TGW at increased risk for these infections. Certain ongoing studies are evaluating doxy PEP and PrEP, including the risk for the development of antimicrobial resistance. The available evidence in the context of increased national incidence of syphilis, chlamydia, and gonorrhea supports consideration of this approach for MSM and TGW at substantial risk for acquiring bacterial STIs. These guidelines will be updated as additional data become available.
